# Ribosome signatures aid bacterial translation initiation site identification

**DOI:** 10.1186/s12915-017-0416-0

**Published:** 2017-08-30

**Authors:** Adam Giess, Veronique Jonckheere, Elvis Ndah, Katarzyna Chyżyńska, Petra Van Damme, Eivind Valen

**Affiliations:** 1 Computational Biology Unit, Department of Informatics, University of Bergen, Bergen, 5020 Norway; 2 VIB-UGent Center for Medical Biotechnology, B-9000 Ghent, Belgium; 3 Department of Biochemistry, Ghent University, B-9000 Ghent, Belgium; 4 Lab of Bioinformatics and Computational Genomics, Department of Mathematical Modelling, Statistics and Bioinformatics, Faculty of Bioscience Engineering, Ghent University, B-9000 Ghent, Belgium; 5 Sars International Centre for Marine Molecular Biology, University of Bergen, 5008 Bergen, Norway

**Keywords:** Ribosome profiling, Bacterial translation initiation, Machine learning, N-terminal proteomics, Proteogenomics

## Abstract

**Background:**

While methods for annotation of genes are increasingly reliable, the exact identification of translation initiation sites remains a challenging problem. Since the N-termini of proteins often contain regulatory and targeting information, developing a robust method for start site identification is crucial. Ribosome profiling reads show distinct patterns of read length distributions around translation initiation sites. These patterns are typically lost in standard ribosome profiling analysis pipelines, when reads from footprints are adjusted to determine the specific codon being translated.

**Results:**

Utilising these signatures in combination with nucleotide sequence information, we build a model capable of predicting translation initiation sites and demonstrate its high accuracy using N-terminal proteomics. Applying this to prokaryotic translatomes, we re-annotate translation initiation sites and provide evidence of N-terminal truncations and extensions of previously annotated coding sequences. These re-annotations are supported by the presence of structural and sequence-based features next to N-terminal peptide evidence. Finally, our model identifies 61 novel genes previously undiscovered in the *Salmonella enterica* genome.

**Conclusions:**

Signatures within ribosome profiling read length distributions can be used in combination with nucleotide sequence information to provide accurate genome-wide identification of translation initiation sites.

**Electronic supplementary material:**

The online version of this article (doi:10.1186/s12915-017-0416-0) contains supplementary material, which is available to authorized users.

## Background

Identification of translated open reading frames (ORFs) is a critical step towards gene annotation and the understanding of a genome. The rapid advances in sequencing have resulted in a deluge of new genomes, making manual annotation intractable and the development of accurate automated methods a necessity. In prokaryotes, ORF delineation is particularly challenging since genes are often tightly packed and frequently overlapping. Whole genome ORF identification in prokaryotes is most commonly performed in silico, using a variety of sequence features, such as guanine-cytosine (GC) codon bias, and motifs, such as the ribosomal binding site or Shine–Dalgarno (SD) sequence [1–3], to differentiate those ORFs that are thought to be functional from those that occur in the genome by chance. While these techniques are able to identify genomic regions containing ORFs with a high accuracy [3], predicting translation initiation sites (TISs), and thus the exact beginning of a protein coding sequence (CDS), is substantially more challenging. In addition to providing functional information via the peptide sequence, regulatory and targeting information are often contained within protein N-termini [4, 5], making accurate identification of the beginning of ORFs essential. This has led to the development of a number of in silico-based TIS identification methods relying on a variety of sequence features [6–9], typically applied after initial ORF annotation in order to re-annotate the often erroneously predicted TIS.

High throughput proteogenomics has the potential to enable identification of protein N-termini, and by extension TISs, from an entire proteome. In practice, however, variation in protein expression levels, physical properties, MS-incompatibility and the occurrence of protein modifications limit the number of detectable protein N-termini [10, 11]. In prokaryotes, N-terminal proteomics typically captures the corresponding peptides of hundreds to the low thousands of genes [11]. For example, a recent study identified N-terminal peptides of 910 of the 4140 (22%) annotated genes in *Escherichia coli* [12]. Although falling short of providing full genome annotation, such datasets provide an effective means of experimental TIS validation.

Significantly higher coverage of TISs can be achieved with sequencing-based technologies. By specifically focusing on ribosome protected reads, ribosome profiling (ribo-seq) [13] infers which parts of the transcriptome are actively undergoing translation. Briefly, ribo-seq aims to capture, select and sequence mRNA reads that are associated with ribosomes, typically reads of 26–34 nt (eukaryotic) [14, 15] or 20–40 nt (prokaryotic) [16, 17] in length. These reads are then commonly assigned to a fixed offset [15, 17–20], or a read length-dependent offset [14, 21, 22], in order to resolve the translated codon represented by each read. In this way, ribo-seq has been used to demonstrate translation of many RNAs and regions that were not thought to be associated with ribosomes [14, 18, 21, 23–26]. Being able to identify translation on a transcriptome-wide scale has obvious application to ORF annotation and a number of methodologies have been developed for prediction of translated ORFs [15, 18, 21, 22, 27]. These methods rely on a number of features, like codon periodicity, read context and read lengths, to distinguish footprints indicative of translation from other, non-translating footprints frequently observed in ribo-seq data. While progress has been made on finding translated regions, delineating their exact boundaries has received less attention. Antibiotic treatment can be used to stall and capture footprints from the initiating ribosome [14, 28, 29], but finding a suitable compound has been elusive in prokaryotes, with only one dataset available to date [30].

**Fig. 1:**
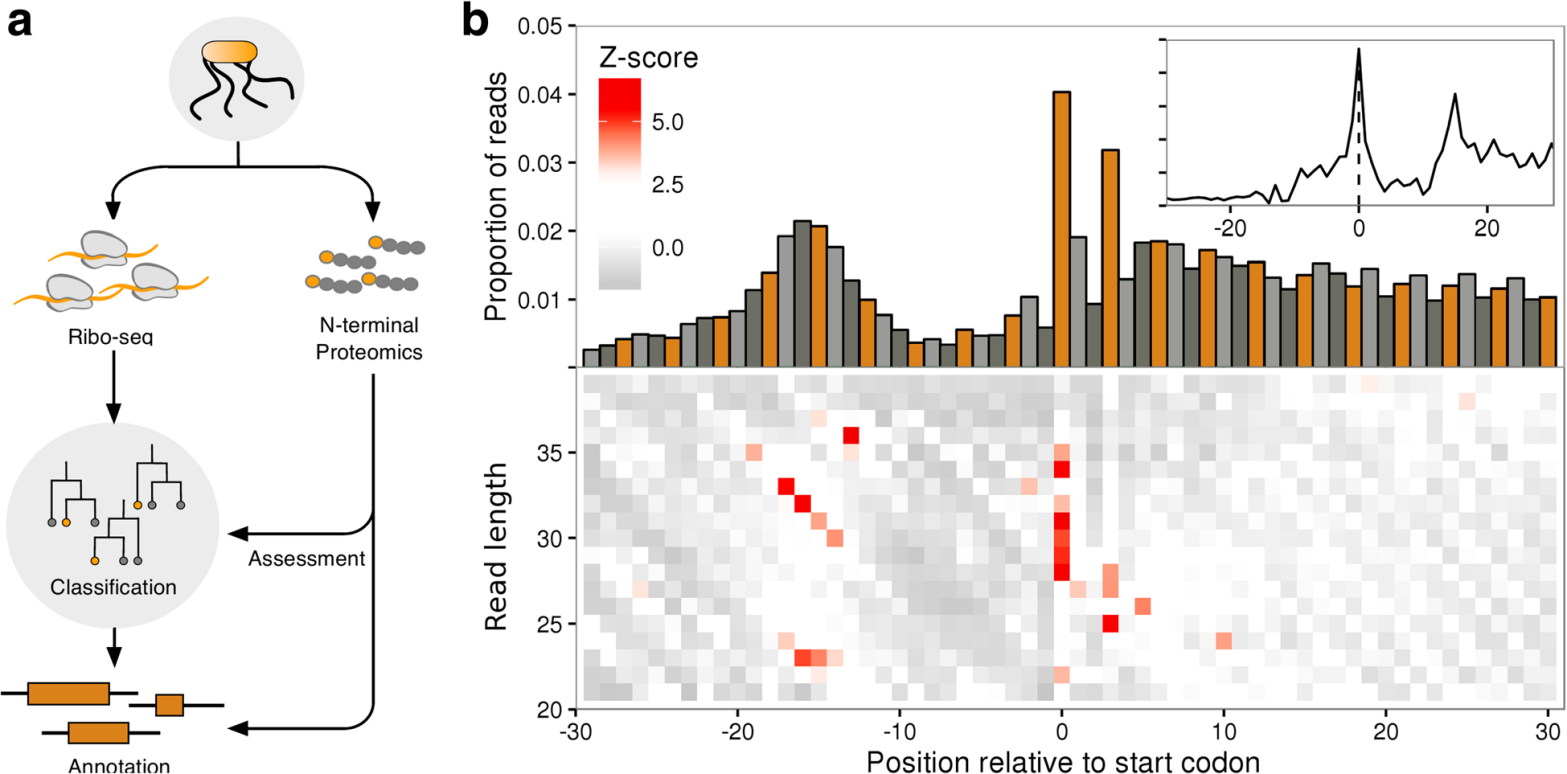
Translation initiation site classification with ribo-seq read length patterns. **a** Schematic representation of the classification strategy. **b** Ribo-seq meta profiles in windows around start codons for all annotated coding sequences in the *S*. Typhimurium genome (monosome sample, n = 4187), contributions from each gene are scaled to a sum of one; (**upper panel**) proportion of 5’ ribo-seq read counts per nucleotide position, coloured by codon position; (**inset**) proportions of ribo-seq read counts per nucleotide position, after adjusting by read length offsets (see methods); (**lower panel**) heatmaps of z-scores of 5’ ribo-seq read counts per read length

Here, we present a generally applicable method that does not depend on specialised chemical treatment, but can take advantage of such data (Fig. 1a). Using N-terminal proteomics we demonstrate its high accuracy and show that it is consistent with other features linked to translation initiation. Applying the model, we predict numerous novel initiation sites in *Salmonella enterica* serovar Typhimurium and several novel genes.

## Results

### Ribosomal signatures of translation initiation

To investigate whether ribo-seq could aid in the accurate delineation of translated ORFs, we generated two ribo-seq libraries from monosome- and polysome-enriched fractions originating from *S*. Typhimurium. The similarities in the profiles of the two libraries (Additional file 1: Figure S1a–d), taken with current literature reports of similarities in the translational properties of polysome and monosome fractions [31], suggest that it is reasonable to consider these libraries sufficiently similar to serve as replicates for the purpose of initiation site prediction. The libraries were initially processed using a standard ribo-seq work-flow [14, 21, 22], trimmed footprints were aligned to a reference genome, and then adjusted based on 5’ read profiles to determine the specific codon under active translation (Fig. 1b, inset). When exploring the processed reads, we discovered that, consistent with previous reports [20, 30], annotated start sites of prokaryotic ribosomes carry a specific signature around the initiating codon (Fig. 1b, inset). Examining the unprocessed reads, we observed that the pattern is a consequence of a specific distribution of read lengths (Fig. 1b), information which is typically lost in pipelines that pre-process the read signal by adjusting reads (Fig. 1b, inset). More specifically, heatmaps of 5’ read profiles indicate that the pattern consists of an enrichment of longer reads (30–35 nucleotides (nt)) starting 14–19 nt upstream of the initiation codon (a diagonal pattern), but ending at the same location, 15 nt downstream of the initiation codon. A shorter set of reads (23–24 nt) are enriched in the same region, but have different end points, 7–9 nt downstream of the initiation codon. Finally, a strong enrichment of 5’ ends of reads of length 28–35 nt can be observed exactly over the start codon itself (Fig. 1b). Looking at the compositions of these patterns, we observed a strong contribution from SD motifs (Additional file 1: Figure S2a, b), apparent as longer reads (30–35 nt) with a fixed 3’ end and a 5’ end dependent on the length of the SD motif from the TIS (Additional file 1: Figure S2c), as reported by O’Connor et al. [16] and Mohammad et al. [17]. Additionally, we discovered a smaller enriched read subset (24–26 nt) for which both 3’ and 5’ ends are dependent on the distance between the SD and the TIS (Additional file 1: Figure S2c). The SD sequence also impacts the distribution of reads immediately downstream, which show a depletion leading up to the TIS (Fig. 1b and Additional file 1: Figure S2 bar charts). Finally, we detected codon-specific enrichments of reads beginning at the first nucleotide of ATG and TTG codons (Additional file 1: Figure S3a, b) and, to a lesser extent, ending at the first codon position in GTG codons (Additional file 1: Figure S3c). These codon-specific enrichments could plausibly originate from experimental artefacts such as sequence-specific ligation or, perhaps more likely, from the sequence-specific digestion biases that have been reported to influence ribo-seq datasets [32, 33].

### Ribosome profiling enables accurate annotation of TISs

**Fig. 2:**
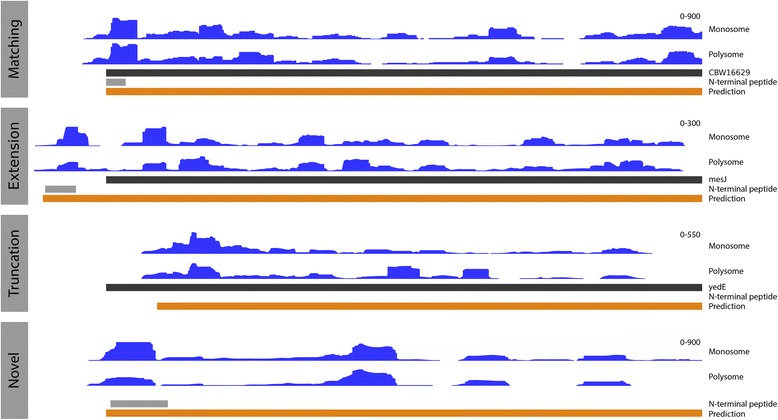
Examples of predicted translated open reading frames (ORFs). Showing genomic tracks of unadjusted ribo-seq read coverage in blue (y axis scale on the right hand side), annotated genes in black, predicted ORFs in orange and N-terminal peptides in grey; (**upper**) a predicted ORF in agreement with the annotated ORFs, supported by ribo-seq coverage and N-terminal evidence; (**middle upper**) a predicted extension relative to the annotated ORF, with N-terminal evidence and ribo-seq coverage supporting the extended prediction; (**middle lower**) a predicted truncation relative to the annotated ORF, with support from ribo-seq coverage; (**lower**) a novel predicted ORF (Chromosome:2064124-2064558), supported by N-terminal evidence and ribo-seq coverage

We trained a random forest model on TISs from the top 50% translated ORFs (see methods) to recognise the patterns in 5’ ribo-seq read lengths and sequence contexts in a –20 to +10 nt window around start codons. In addition, we encoded information about the start codon position within the ORF and the read abundance upstream and downstream of the start sites. The model was used to predict TISs from all in-frame cognate and near cognate start codons around annotated genes in the *S*. Typhimurium genome. Predictions on the two samples were highly accurate, with area under the curve (AUC) values of 0.9958 and 0.9956 on independent validation sets for the monosome and polysome samples, respectively (parameter importance for the models is summarised in Additional file 2: Tables S1, 2). In total, 4610 (monosome) and 4601 (polysome) TISs were predicted in the two sets. From these, we constructed a high confidence set from predictions common to both replicates. In total, this set contained 4272 predictions, representing an 86.50% agreement between the replicates. The discrepancies predominantly originate from genes with limited translation. Of the high confidence TISs, 3853 matched annotated ORFs, 214 represented extensions and 205 truncations. Representative examples of predicted extended, truncated and matching ORFs are shown in Fig. 2.

**Fig. 3:**
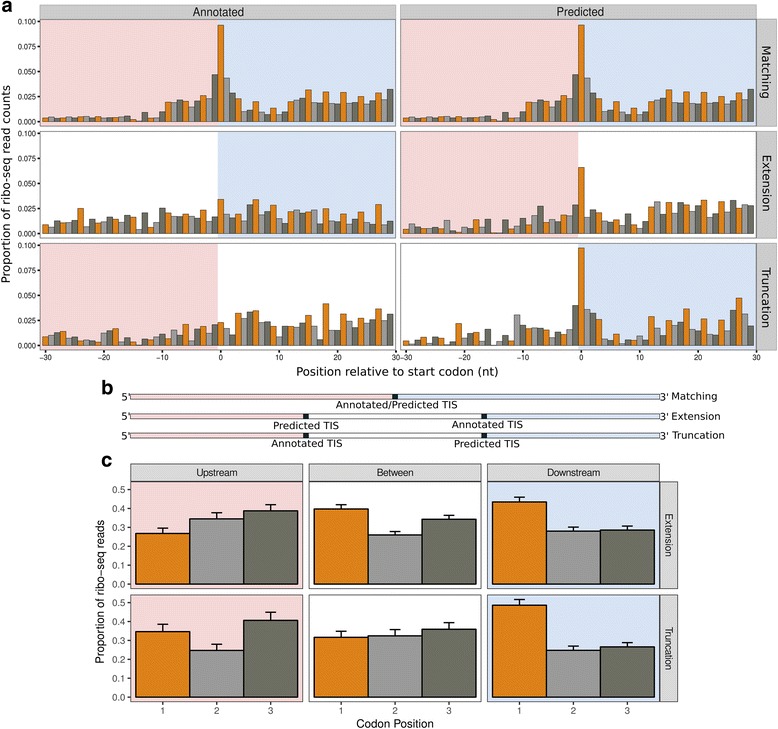
Ribo-seq reads and periodicity are consistent with re-annotated translation initiation sites. Bar colour indicates codon position. Downstream regions are highlighted in pink, upstream regions are highlighted in light blue. **a** Meta-plots showing the proportion of scaled ribo-seq reads, after read length-specific adjustment, in relation to annotated or predicted translation initiation sites, for open reading frames matching annotated genes (n = 3853), predicted extensions (n = 214) or predicted truncations (n = 205). Contributions from each gene are scaled to a sum of one. Annotated translation initiation sites (TISs) show statistically significant increases in ribo-seq density upstream (extensions, Wilcoxon rank sum test W = 252,580, *P* = 2.156 × 10^–6^), or downstream of start codons (truncations, Wilcoxon rank sum test W = 293,200, *P* = 1.139 × 10^–5^). **b** Transcript models. **c** Bar plots with standard error of the mean, showing the proportion of scaled ribo-seq read counts, after read length-specific adjustment, in each codon position. For truncations, regions are 30 nt upstream of the annotated TIS, between the annotated and predicted TIS, and 30 nt downstream of the predicted TIS. For extensions, regions are 30 nt upstream of the predicted TIS, between the predicted and annotated TIS, and 30 nt downstream of the annotated TIS; 3 nt periodicity does not occur upstream of predicted TISs (truncations), but does occur upstream of annotated TISs (extensions)

As expected, the predictions show the same start codon usage distribution (Additional file 1: Figure S4a) and carry the same read distribution signature as the annotated sites (Additional file 1: Figure S4b). Consistent with annotated initiation sites, an increase in ribosome protected reads can be seen downstream versus upstream of the predicted TIS (Fig. 3a). Furthermore, extended ORFs exhibit a shift in ribo-seq density downstream relative to the annotated TIS, consistent with the predicted extension. Conversely, truncated ORFs exhibit a shift in read density upstream relative to the annotated TIS and consistent with the predicted truncation.

**Fig. 4:**
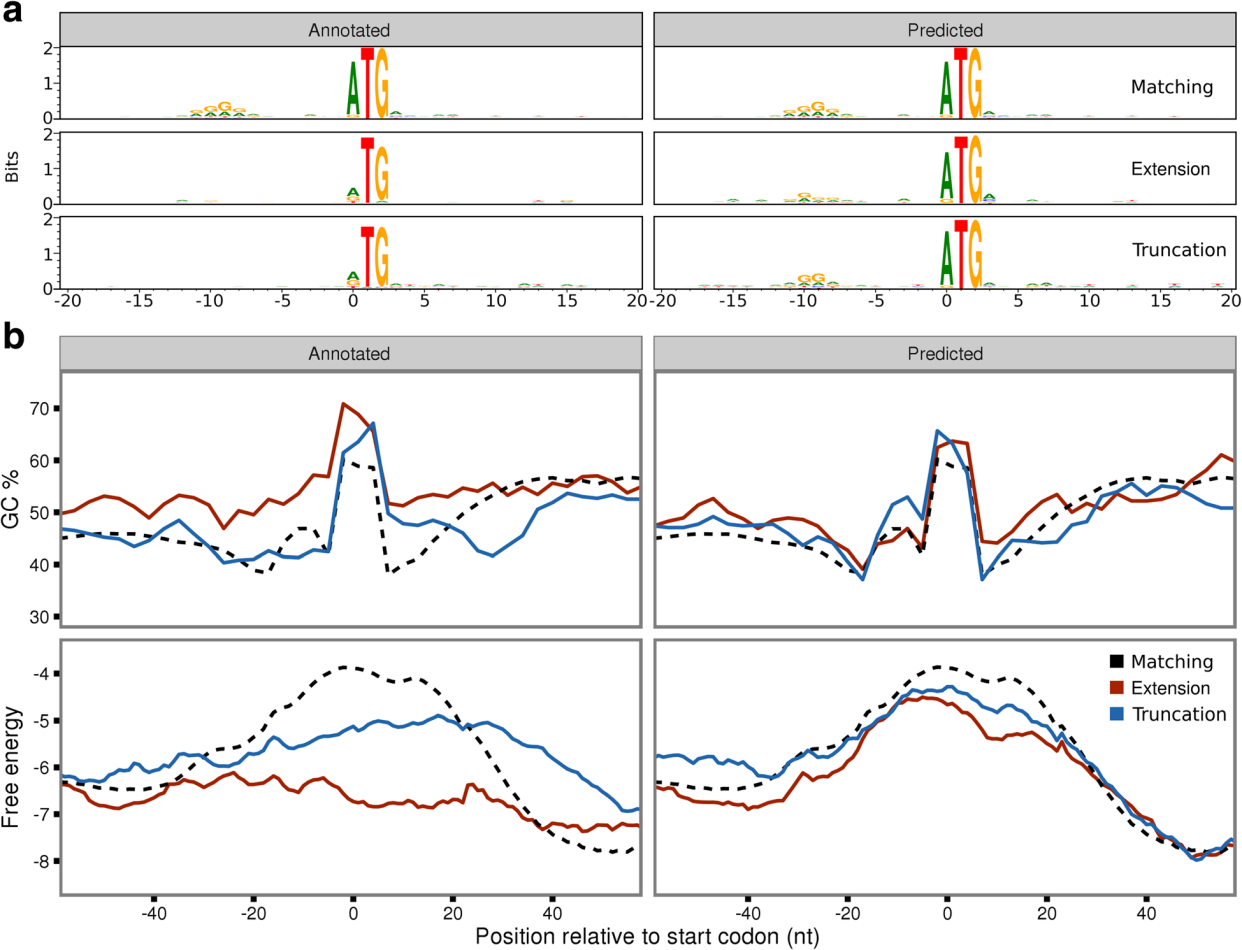
Sequence and structure features support re-annotation of translation initiation sites (TISs). **a** Sequence motifs relative to annotated or predicted TISs in the same genes. ‘Matching’ (n = 3853) are identical, while predicted extensions (n = 214) and truncations (n = 205) have stronger Shine-Dalgarno sequences than their annotated counterparts. **b** Meta-profiles relative to annotated or predicted TISs, with lines representing open reading frames matching annotated genes (dashed black), predicted extensions (red) and predicted truncations (blue). (**upper**) Mean guanine-cytosine (GC) content at third codon positions, averaged over 9 nt sliding windows. Predicted TISs match the expected profile more closely than annotated positions (Wilcoxon rank sum test W = 463,640, *P* = 0.001665 for extensions, W = 453,510, *P* = 0.0001546 for truncations), showing an increase in GC content immediately after the start codon, whereas annotated extensions and truncations are less similar to the expected profile (Wilcoxon rank sum test W = 493,250, *P* = 1.226 × 10^–9^ for extensions, Wilcoxon rank sum test W = 460,810, *P* = 5.395 × 10^–5^ for truncations), showing shifts down or upstream in annotated TIS. Peaks over the zero position correspond to nucleotide biases in start codon selection. (**lower**) Meta-profiles of mean free energy averaged in 39 nt sliding windows. Peaks of low secondary structure potential, expected to occur over start codons, are centred over predicted TIS, but are clearly shifted down or upstream of annotated TIS, in predicted extensions and truncations

To further assess the predictions we compared the newly predicted TISs with the previously, potentially erroneously, annotated TIS. A highly significant sequence feature of translation initiation sites is the SD sequence, which facilitates translation initiation in prokaryotes [34]. The consensus sequence GGAGG is located approximately 10 nt upstream of the start codon [35]. The predicted initiation sites show clear evidence of SD sequences centred 9–10 nt upstream of the start codon (Fig. 4a). Strikingly, the annotated TISs, in the same genes where our model has predicted novel sites, show an absence of the SD sequence (Fig. 4a). Since our model considers sequence context and SD-associated profiles, it is unsurprising that the predictions carry this signature, but the absence of these motifs around previously annotated start codons is notable.

Besides the presence of SD sequences, the GC content is commonly used to identify CDSs in prokaryotes. The overall GC content of a genome or genomic region is often highly optimised. In coding regions, this optimisation can be achieved via synonymous substitutions, predominantly at third codon positions [36], a finding explaining the pronounced bias in the GC content of third nucleotide positions observed in coding regions compared to the rest of the genome [37, 38]. Interestingly, at those annotated sites where our model predicts an extension, an increase in GC content upstream of the annotated start codon can clearly be observed consistent with the presence of an initiation site further upstream. Conversely, predicted truncations show a decrease downstream of the annotated start codon. In contrast, at predicted sites, both predicted extensions and truncations fit closely to the expected distribution (Fig. 4b upper). While our model has some potential to capture GC bias (at the nucleotides in positions –20 to +9 nt), the observable shifts in GC content, in relation to the annotated TIS where the model predicts an alternative TIS, argue against the validity of the annotated TIS.

Another significant feature of prokaryotic translation initiation is the absence of intrinsic structure in the region around the start codon, enabling easier access for ribosomes to bind [39]. We therefore calculated the average free energy over all predicted sites and compared them to the previous annotation in the same genes. Consistent with GC content patterns, ORFs where we predict an extension or truncation show lower free energy over the TIS at the previously annotated positions. For truncated ORFs, we also observe a higher propensity to form secondary structure downstream of the annotated start codon. In the predicted sites, these less-structured regions can clearly be observed directly over the start codon, highly indicative of true initiation sites (Fig. 4b lower).

Ribosomes translocate along mRNAs three nucleotides at a time, corresponding to one codon and an amino acid. Consequently, reads originating from bona fide translated regions also exhibit a three nucleotide periodicity in adjusted read counts, with a bias towards mapping to the first nucleotide in each codon [22]. At initiation sites, the read distribution therefore switches from a random distribution upstream to a periodic, biased distribution downstream, as demonstrated in Additional file 1: Figure S1a–d. While it has been argued that periodicity of ribosome profiling reads in prokaryotic genomes can be caused by the third codon GC bias (described above) [33], we observe that the periodicity is independent of third codon GC content (Additional file 1: Figure S5). Comparing the density of reads falling into each of the three codon positions, in extended ORFs, we observe increased read density at the first nucleotide position upstream of annotated, but not of predicted, TISs. Similarly, at truncated ORFs we see a decrease in the density of reads at the first nucleotide position downstream of the annotated TIS (Fig. 3c, “Between”), but not downstream of predicted TIS (Fig. 3c, “Downstream”).

Taken together, the patterns in read distribution, SD motifs, GC bias, unstructured regions and three nucleotide periodicity, provide clear and consistent support that the TISs, which we re-annotate, show, on average, a higher agreement with features indicative of canonically translated prokaryotic ORFs, than their corresponding previously annotated counterparts.

### N-terminal proteomics confirms predicted sites

**Fig. 5:**
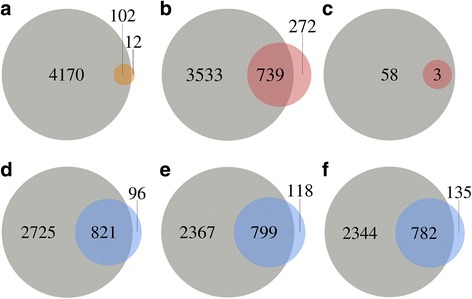
Predicted open reading frames (ORFs) show high agreement with validation datasets. Venn diagram showing the agreement of predicted *S*. Typhimurium ORFs with (**a**) high confidence N-terminal peptides (orange) or (**b**) blocked N-terminal peptides (red), novel predicted *S*. Typhimurium ORFs (**c**) with blocked N-terminal peptides supporting novel ORFs (red) and predicted ORFs from the *E. coli* tetracycline (**d**), Li ﻿et al. (**e**) or Mohammad ﻿et al. (**f**) datasets with ecogene verified protein starts (blue)

In order to experimentally validate the accuracy of the predictions, positional proteomics analyses enriching for protein N-termini were performed. Blocked N-termini were identified matching 1040 *S*. Typhimurium ORFs, from which a high confidence subset of Nt-formylated initiator methionine (iMet)-starting N-termini was selected (see methods) and used to assess the accuracy of the model. In total, 114 high confidence N-termini were identified, supporting 102 annotated CDSs, 3 N-terminal CDS extensions and 9 N-terminal CDS truncations. Because genomic positions with N-terminal peptide support were excluded from the set used to train the random forest model, these high confidence TIS positions can be used to determine the accuracy of the predictions. Of the 102 N-terminally supported annotated genes, 97 were predicted by the model. Furthermore, two of the extensions and four of the truncations were captured (Fig. 5a, Additional file 2: Table S3). Assuming that none of these genes had multiple initiation sites, the sensitivity, specificity and positive predicted value of the model were estimated to be 0.9450, 0.9993 and 0.9537, respectively.

We found blocked N-terminal peptides matching the predicted start positions from three distinct novel regions (defined as ORFs at least 300 nt in length, in regions that were not overlapping with annotated genes or regions at least 999 bp upstream of annotated genes). Comparing the predictions to the blocked N-termini identified, we found support for 694 predictions that match annotated TISs, 23 predicted extensions, 22 predicted truncations and 3 novel ORFs (Fig. 5b, c, Additional file 2: Table S4).

In order to determine the contribution of sequence and ribosome read length features to the predictive performance, models were trained without including either of these feature sets or with just one of these feature sets. When either the ribo-seq read length or sequence information is excluded from the model, we observed a decrease in sensitivity (0.8785 and 0.3364 when excluding ribo-seq read lengths or sequence features, respectively, compared to 0.9450 when all features are used), while maintaining high specificity (0.9990 in both cases) (Additional file 2: Table S5). We observed that sequence features alone were able to correctly identify 85 of the 114 high confidence N-terminally supported TISs, and that ribo-seq read length information alone was able to correctly identify 36 of the 114 high confidence N-terminally supported TIS. Although sequence information had a larger impact on sensitivity than ribo-seq read lengths, the optimal values were only achieved when both sequence and read length features were used in combination (Additional file 2: Table S5).

### TISs are predicted at novel genomic regions

In order to discover potential novel translated ORFs, we applied our prediction models to look for TISs in genomic regions outside annotated ORFs. Novel ORFs that were similar in size to known CDSs (>100 amino acids) and with ribo-seq coverage along a high proportion of the ORF (>75% coverage, see methods) were considered candidate translated novel ORFs. Of the 219 (monosome) and 193 (polysome) ORFs under consideration, 104 and 115 novel translated ORFs were predicted, respectively; 61 of these novel translated ORFs were common to both replicates (38.61% agreement) and used as a high confidence set of novel predictions. Unlike the annotated genes, these novel ORFs were not previously confirmed as translated regions and most had a significantly lower read density (mean fragments per kilobase per million mapped reads (FPKM) of 8) than annotated genes (mean FPKM of 126). The higher discrepancy between the two replicates is mainly a consequence of low-abundance ORFs that did not pass the coverage threshold in either of the replicates.

Read density plots over the novel ORFs revealed features consistent with protein coding regions, but with higher variance due to the low number of ORFs. Specifically, GC content increases downstream of the initiation codon, the regions around the initiation codon have less intrinsic structure potential and SD sequences were present upstream (Additional file 1: Figure S6). Additionally, three of the predicted novel translated ORFs were supported by N-terminal peptide evidence (Fig. 5c) (a representative example is shown in Fig. 2). A further 22 newly predicted ORFs showed high similarity to known protein sequences, four of which contained functional protein domains (Additional file 2: Table S6).

### Tetracycline-treated samples improve classifier accuracy

While reads isolated from elongating ribosomes provide sufficient information to predict the majority of translation start sites, we set out to explore the full potential of our classifier in combination with data from initiating ribosomes. A recent study on *E. coli* [12] demonstrated the use of tetracycline as a translation inhibitor to enrich for footprints from initiating ribosomes in prokaryotes. Herein, the tetracycline datasets showed the expected pattern from initiating ribosomes as a range of read lengths starting 28–14 nt upstream of the initiation codon (5’ data), but ending at the same positions 14–15 nt downstream of the initiation codon (3’ data). An additional pattern of shorter read lengths was also observed, starting 26–18 nt upstream and ending 2 nt downstream of the initiation codon (Additional file 1: Figure S7a, b). Complementary datasets were selected from publically available *E. coli* ribo-seq libraries collected via flash freezing [17, 40]; in these datasets, the pattern was formed by an enriched set of reads lengths beginning 14 to 23 nt upstream of the initiation codon, ending directly at the initiation codons, and by a secondary set of read lengths starting at the initiation codons and ending 19–39 nt downstream (Additional file 1: Figure S7c–f).

We trained separate classifiers on tetracycline (initiating) libraries and flash frozen (non-specific) datasets, using two replicates for each dataset (Additional file 2: Table S7). Model performance was assessed with receiver operating characteristic curves using a validation dataset (see methods) for each replicate. The resulting AUC values of 0.9993 and 0.9994 in the tetracycline replicates were comparable to those of the flash frozen libraries (Li: 0.9998 and 0.9997, Mohammad: 0.9998 and 0.9993). The parameter importance in each of the models is shown in Additional file 2: Tables S8–S13. In the tetracycline dataset, a total of 3711 ORFs were predicted, with 86 extensions and 79 truncations (Additional file 2: Table S14). The flash frozen models predicted a total of 3269 and 3341 ORFs, including 48 and 73 extensions and 95 and 102 truncations in the Li and Mohammad libraries, respectively (Additional file 2: Table S15, S16).

*E. coli* predictions were assessed against the ecogene curated set of 923 experimentally verified protein starts [41]. Genes within this dataset were excluded from the sets used to train the random forest models in order to provide a means of assessing the accuracy of the ORF predictions. Five of the verified protein starts corresponded to pseudogenes without annotated CDSs; of the remaining 917 verified protein starts, 821 (89.53%) matched ORFs from the tetracycline predictions, with 24 (2.62%) predicted ORFs in disagreement with the curated set (11 extensions, 13 truncations) (Fig. 5d, Additional file 2: Table S14). In the Li flash frozen predictions, 782 (85.28%) were found to match ecogene start sites and 26 (2.84%) were found to be inconsistent (7 extensions, 18 truncations) with the verified protein starts (Fig. 5e, Additional file 2: Table S15). Of the Mohammad flash frozen predictions, 779 (84.95%) were found to match and 29 (3.16%) were found to disagree (12 extensions, 17 truncations) with ecogene verified protein starts (assuming genes do not have multiple TISs) (Fig. 5f, Additional file 2: Table S16). Based on the experimentally verified starts, the tetracycline-based classifier resulted in higher accuracy (sensitivity 0.9194, specificity 0.9996, positive predictive value 0.9716) than either of the flash frozen-based classifiers (sensitivity 0.8777/0.8773, specificity 0.9996/0.9995, positive predictive value 0.9678/0.9641 (Li/Mohammed)). Surprisingly, the difference was not substantial, arguing that using initiating ribosomes is not a prerequisite to obtain a good annotation of initiation sites.

## Discussion

Our model shows that the distribution of ribo-seq footprint lengths can be used in conjunction with sequence features to accurately determine the translation initiation landscape of prokaryotes. These patterns are typically disrupted in standard ribo-seq analysis when reads are adjusted and merged to determine the specific codon under translation. The model is applicable across multiple organisms and experimental conditions, and can be augmented with data from initiating ribosomes. It exhibits high accuracy as assessed by cross-validation, N-terminal proteomics and independent sequence-based metrics such as potential to form RNA structures. Interestingly, the predicted TISs exhibited known features of translation initiation, while the previously matching annotations do not. In *S*. Typhimurium, our model provides evidence for 61 novel translated ORFs and the re-annotation of 419 genes. In particular, the current annotation includes 19 genes that lacked initiation codons, of which we were able to re-annotate 15 (Additional file 2: Table S4).

As expected, models based on initiating reads performed better than models based on non-specific ribo-seq reads, suggesting that an optimal strategy for TIS identification would favour the use of the more focused, initiating ribo-seq profiles. However, the degree of improvement between the models was relatively small, confirming the suitability of both non-specific and initiating ribo-seq libraries for the purposes of TIS and ORF detection.

Footprints containing SD sequences have been shown to produce longer ribo-seq reads, attributed to nuclease protection from RNA/anti-SD interactions [16, 17]. We observed enrichments of these longer read lengths at footprints overlapping the SD, but also found a second population of shorter SD associated reads, less prominent at internal SD sequences than those upstream of TISs (Additional file 1: Figure S2). It is interesting to note the importance the models place (Additional file 2: Tables S1, S2) on this shorter range of reads (23–25 nt) in the *S*. Typhimurium samples (Additional file 1: Figure S8). These shorter reads were also consistent with recent reports of ribosomal subunits in a variety of distinct configurations, observed from translation complex profiling in the eukaryote *Saccharomyces cerevisiae* [42]. Whether similar patterns of read length distributions can be observed in eukaryotic ribo-seq datasets remains to be determined, although conceptually the method and metrics described herein are fully extendable to eukaryotic datasets.

A key strength of this approach is that the model is able to build complicated rules incorporating multiple sets of features from changes in ribosome footprint density, sequence context and ribosomal profiling patterns indicative of RNA/ribosome interactions and initiation codons. Leaderless genes, for example, might not be expected to have an SD motif, but the flexibility in our model would allow these to be identified if other features were suggestive of TISs. For example, 3387 of the *S*. typhimurium TISs predicted do not have a strong SD sequence (defined as binding energy of ≤ –8 kcal/mol, see methods).

While the model relies on some pre-existing annotated ORFs for training, it does not require any prior knowledge, but rather detects the RNA/ribosome interactions of SD and initiation sites from patterns in the fragment length of protected reads. This may provide a fruitful avenue for exploring novel sequence features, for example, using patterns in protected read lengths as a proxy to identify ribosome/RNA interactions in species with alternative ribosome binding motifs or initiation mechanisms.

## Conclusions

In conclusion, this study demonstrates the utility of ribo-seq fragment length patterns for TIS identification across multiple experimental conditions. These models provide a significant step forward in experimental TIS discovery, facilitating the move towards complete ORF annotation in both presumably well-annotated model organisms, as well as the ever growing list of newly sequenced genomes.

## Methods

### Preparation of ribo-seq libraries

Overnight stationary cultures of wild type *S*. Typhimurium (*Salmonella enterica* serovar Typhimurium, strain SL1344) grown in LB media at 37 °C with agitation (200 rpm) were diluted at 1:200 in LB and grown until they reached and OD600 of 0.5 (i.e. logarithmic (Log) phase grown cells). Bacterial cells were pre-treated for 5 min with chloramphenicol (Sigma Aldrich) at a final concentration of 100 μg/mL prior to collection by centrifugation (6000 × *g*, 5 min) at 4 °C. Collected cells were flash frozen in liquid nitrogen. The frozen pellet of a 50 mL culture was re-suspended and thawed in 1 mL ice-cold lysis buffer for polysome isolation (10 mM MgCl_2_, 100 mM NH_4_Cl, 20 mM Tris-HCl pH 8.0, 20 U/mL of RNase-free DNase I (NEB 2 U/μL), 1 mM chloramphenicol (or 300 μg/mL), 20 μL/mL lysozyme (50 mg/mL in water) and 100 μ/mL SUPERase.In™ RNase Inhibitor (Thermo Fisher Scientific, Bremen, Germany)), vortexed and left on ice for 2 min with periodical agitation. Subsequently, the samples were subjected to mechanical disruption by two repetitive cycles of freeze-thawing in liquid nitrogen, and 5 mM CaCl_2_, 30 μL 10% DOC and 1 × complete and EDTA-free protease inhibitor cocktail (Roche, Basel, Switzerland) were added and the mixture was left on ice for 5 min. Lysates were clarified by centrifugation at 16,000 × *g* for 10 min at 4 °C.

For the monosome sample, the supernatant was subjected to MNase (Roche Diagnostics, Belgium) digestion using 600 U MNase (~1000 U per mg of protein). Digestion of polysomes proceeded for 1 h at 25 °C with gentle agitation at 400 rpm and the reaction was stopped by the addition of 10 mM EGTA. Next, monosomes were recovered by ultracentrifugation over a 1 M sucrose cushion in polysome isolation buffer without RNase-free DNase I and lysozyme, and by the addition of 2 mM DTT using a TLA-120.2 rotor for 4 h at 75,000 rpm and 4 °C.

For the selective purification of monosomes from polysomes (polysome sample), the supernatant was resolved on 10–55% (w/v) sucrose gradients by centrifugation using an SW41 rotor at 35,000 rpm for 2.5 h at 4 °C. The sedimentation profiles were recorded at 260 nm and the gradient fractionated using a BioComp Gradient Master (BioComp) according to the manufacturer’s instructions. Polysome-enriched fractions were pooled and subjected to MNase digestion and monosome recovery, as described above.

Ribosome-protected mRNA footprints, with sizes ranging from 26 to 34 nt, were selected and processed as described previously [14], with some minor adjustments [43]. The resulting ribo-seq cDNA libraries of the monosome and polysome sample were duplexed and sequenced on a NextSeq 500 instrument (Illumina) to yield 75 bp single-end reads.

### Ribo-seq data processing

Ribo-seq data were pre-processed with cutadapt (version 1.9.1) [44] to remove sequencing adaptors, discarding reads less than 20 nt in length after trimming. Trimmed reads were initially aligned to the SILVA RNA database version 119 [45], the remaining reads were then mapped to either *S. enterica* serovar Typhimurium, strain SL1344 (Assembly: GCA_000210855.2) or *E. coli* str. K-12 substr. MG1655 (Assembly: GCA_000005845.2). Alignments were performed with bowtie2 (version 2.2.4) [46]. Reads were brought to codon resolution by adjusting the 5’ position of each read by a fixed distance offset, specific to each read length, based on visual identification of periodicity meta plots of the read counts per read length (Additional file 1: Figure S9). In the *S*. Typhimurium dataset the following read lengths were selected and adjusted by the values in brackets, in the monosome sample 29 (13 nt), 30 (14 nt), 31 (15 nt), 32 (16 nt), 33 (17 nt), and in the polysome sample 29 (13 nt), 30 (14 nt), 31 (15 nt), 32 (16 nt), 33 (17 nt) and 34 (18 nt). Selected reads of the indicated lengths account for 39.98% and 48.69% of total reads for the monosome and polysome samples, respectively.

Recent publications reporting prokaryotic ribo-seq [12, 17, 20, 47] suggest that reads from libraries digested with micrococcal nuclease align more precisely to their 3’ rather than 5’ ends. Consistent with this, we observed a modest increase in the periodicity of meta profiles of the *S*. Typhimurium ribo-seq libraries when reads were brought to codon resolution from the 3’ end (Additional file 1: Figure S1a–d); however, this did not hold true for the tetracycline *E. coli* datasets, where the use of 3’ poly adenosine adaptors resulted in a loss of resolution at the 3’ end after read trimming (Additional file 1: Figure S7a, b), making the use of 5’ ends preferable. Since the protected read length patterns used in the input feature vectors for the classifier take both length and position into consideration, the classifier is unaffected by the alignment choice for generating positional data. However, to maintain consistency throughout this study, read counting for model predictors was performed using the 5’ alignments, while the periodicity plots, which are sensitive to read terminus choice, were calculated from either 3’ alignments or from ribo-seq reads after read length-specific read adjustment.

### Read distributions and heatmaps

Ribo-seq read distributions were summarised over all annotated start codons in the *S*. Typhimurium and *E. coli* annotations, respectively. 5’ read counts were taken from regions 30 nt upstream to 60 nt downstream of the start codon (or –100 to –10 nt upstream of the stop codon), 3’ read counts were taken from the first nucleotide of the start codon up to 90 nucleotides downstream (or –70 upstream to 20 nt downstream of the stop codon). All reads with a MAPQ greater than 10, from the upper 90% of genes by total CDS expression, were included. Total counts were scaled to a sum of one per individual region, in order to not disproportionately favour profiles from highly expressed genes. Meta plots were then produced to show the proportion of read counts over the window across all genes. 3’ and 5’ heatmaps were generated from the scaled regions, showing the number of standard deviations from the row (read length) mean. ATG, GTG and TTG codons were taken from in-frame CDS regions, excluding annotated and predicted start codons. SD motifs were identified as sequences predicted to have a binding energy with the anti-SD sequence (AGGAGGTG) of –8 kcal/mol or lower. Energies were calculated in 8 nt overlapping windows across the whole genome with RNAsubopt (version 2.1.9) [48], and assigned to the first “A” of the anti-SD sequence. Upstream SD sequences were defined as those within 30 nt upstream of an annotated start codon. Downstream SD sequences, were defined as SD motifs within annotated CDS regions, excluding those within 50 nt of the start codon of a downstream annotated or predicted ORF. Third codon position GC content was calculated from nucleotide sequences 60–150 nt downstream of the start codon for the upper 90% of genes by total CDS expression. Three prime read distributions were plotted for the upper and lower 10% of sequences based on total third position GC content.

### Model implementation

For each candidate TIS, a feature vector was defined as each nucleotide in a –20 to +10 nt window around the position, the ribo-seq 5’ FPKM between the current position and the next in-frame downstream stop codon, the count of in-frame cognate and near-cognate start sites from the nearest in-frame upstream stop codon to the current position, the proportion of 5’ ribo-seq reads upstream in a 20 nt window, the proportion of 5’ ribo-seq reads downstream in a 20 nt window, the ratio of 5’ ribo-seq reads up and downstream, and the proportion 5’ ribo-seq counts per read length for a fixed range of positions in relation to current site (selected from visual inspection of 5’ read length heatmaps (Additional file 1: Figure S1, S7)). In the *S*. Typhimurium samples, read lengths of 20–35 nt in positions –20 to –11 and 0 nt, were used. In the *E. coli* datasets for the tetracycline samples, read lengths of 20–35 nt at positions –25 to –16 nt were selected. Finally, for flash frozen samples, lengths of 20–35 nt at positions –20 to –11 and 0 nt were used. Stop-to-stop windows were defined for each annotated gene as all in-frame positions between the nearest in-frame upstream stop codon and the stop codon of the gene (with a maximum length cut-off 999 nt upstream).

The H2O random forest implementation (version 3.10.4.6) [49] was used and the models were trained with positive examples of randomly selected annotated start codons from the upper 50% of genes ranked by ribo-seq expression over the gene CDS. We additionally required that the positive examples were not among the genes supported by N-terminal peptides in the *S*. Typhimurium samples or included in the ecogene dataset for the *E. coli* samples, since these were retained for model accuracy assessment. Negative examples were randomly selected from in-frame codons in the stop-to-stop windows both upstream and downstream of the annotated TIS. The *S*. Typhimurium models were trained on 1500 positive and 6000 negative positions, with an independent validation set of 200 positive and 800 negative positions. Parameter tuning was performed for the number of trees in the random forest, using values from 50 to 1000 with a step size of 50, and selecting the value which produced the highest AUC values on the validation set (monosome: 600, polysome: 600) (see code for an example of automated parameter tuning). The *E. coli* models were trained on 1100 positive and 4400 negative positions, with an independent validation set of 200 positive and 800 negative positions for parameter tuning (number of trees: Li1: 550, Li3: 450, Mohammad1: 600, Mohammad2: 700, TET2: 650 and TET3: 700). Predictions were then run against all cognate and near cognate in-frame positions, in the stop-to-stop regions. Novel predictions were performed against all cognate and near cognate codons in stop-to-stop regions around ORFs of at least 300 nt in length, with a ribo-seq read coverage of 0.75 or more (ORF coverage was defined as the proportion of nucleotides in each predicted ORF that at least one ribo-seq read mapped to), that did not overlap with annotated CDSs. ORFs were delineated by extending each candidate TIS to the closest in-frame stop codon. For a given stop-to-stop region the model selected the TIS with the highest positive predicted score per sample. Predictions from the replicates for each of the datasets were then compared, discarding predictions that were unique to only one replicate.

### N-terminal proteomics

Overnight stationary cultures of wild type *S*. Typhimurium (*S. enterica* serovar Typhimurium, strain SL1344) grown in LB media at 37 °C with agitation (200 rpm) were diluted at 1:200 in LB and grown until they reached an OD600 of 0.5 (i.e. logarithmic (Log) phase grown cells). Bacterial cells were collected by centrifugation (6000 × *g*, 5 min) at 4 °C, flash frozen in liquid nitrogen and cryogenically pulverized using a pestle and mortar cooled with liquid nitrogen. The frozen pellet of a 50 mL culture was re-suspended and thawed in 1 mL ice-cold lysis buffer (50 mm NH_4_HCO_3_ (pH 7.9) supplemented with a complete protease inhibitor cocktail tablet (Roche Diagnostics GmbH, Mannheim, Germany) and subjected to mechanical disruption by two repetitive freeze-thaw and sonication cycles (i.e. 2 minutes of sonication on ice for 20-s bursts at output level 4, with a 40% duty cycle (Branson Sonifier 250; Ultrasonic Convertor)). The lysate was cleared by centrifugation for 15 min at 16,000 × *g* and the protein concentration measured using the protein assay kit (Bio-Rad) according to the manufacturer’s instructions. The guanidine hydrochloride (4 M f.c.) was added to the lysate and subjected to N-terminal COFRADIC analysis, as described previously [50]. Free amines were blocked at the protein level making use of an N-hydroxysuccinimide ester of (stable isotopic encoded) acetate (i.e. NHS esters of ^13^C_2_D_3_ acetate), which allows the distinction of in vivo and in vitro blocked N-terminal peptides [51]. The modified protein sample was digested overnight with sequencing-grade modified trypsin (1/100 (w/w trypsin/substrate)) at 37 °C and subsequent steps of the N-terminal COFRADIC procedure were performed as previously described [50].

### LC-MS/MS analysis

LC-MS/MS analysis was performed using an Ultimate 3000 RSLC nano HPLC (Dionex, Amsterdam, the Netherlands) connected in line to an LTQ Orbitrap Velos mass spectrometer (Thermo Fisher Scientific, Bremen, Germany). The sample mixture was loaded on a trapping column (made in-house, 100 μm ID × 20 mm, 5 μm beads C18 Reprosil-HD, Dr Maisch). After back flushing from the trapping column, the sample was loaded on a reverse-phase column (made in-house, 75 m ID × 150 mm, 5 μm beads C18 Reprosil-HD, Dr Maisch). Peptides were loaded in solvent A’ (0.1% trifluoroacetic acid, 2% acetonitrile) and separated with a linear gradient from 2% solvent A” (0.1% formic acid) to 50% solvent B’ (0.1% formic acid and 80% acetonitrile) at a flow rate of 300 nL/min followed by a wash reaching 100% solvent B’. The mass spectrometer was operated in data-dependent mode, automatically switching between MS and MS/MS acquisition for the 10 most abundant peaks in a given MS spectrum. Full scan MS spectra were acquired in the Orbitrap at a target value of 1 × 10^6^ at a resolution of 60,000. The 10 most intense ions were then isolated for fragmentation in the linear ion trap, with a dynamic exclusion of 20 s. Peptides were fragmented after filling the ion trap at a target value of 1 × 10^4^ ion counts. Mascot Generic Files were created from the MS/MS data in each LC run using the Mascot Distiller software (version 2.5.1.0, Matrix Science, www.matrixscience.com/Distiller.html). To generate these MS/MS peak lists, grouping of spectra was allowed with a maximum intermediate retention time of 30 s and a maximum intermediate scan count of 5. Grouping was performed with a 0.005 Da precursor tolerance. A peak list was only generated when the MS/MS spectrum contained more than 10 peaks. There was no de-isotoping and the relative signal-to-noise limit was set at 2.

The generated MS/MS peak lists were searched with Mascot using the Mascot Daemon interface (version 2.5.1, Matrix Science). Searches were performed using a 6-FT database of the *S*. Typhimurium genome combined with the Ensembl protein sequence database (assembly AMS21085v2 version 86.1), which totalled 139,408 entries after removal of redundant sequences. The 6-FT database was generated by traversing the entire genome across the six reading frames and searching for all NTG (N = A, T, C, G) start codons and extending each to the nearest in frame stop codon (TAA, TGA, TAG), discarding ORFs less than 21 nt in length. The Mascot search parameters were set as follows: heavy acetylation at lysine side-chains (Acetyl:2H(3)C13(2) (K)), carbamidomethylation of cysteine and methionine oxidation to methionine-sulfoxide were set as fixed modifications; and formylation, acetylation and heavy acetylation of N-termini (Acetyl:2H(3)C13(2) (N-term)) and pyroglutamate formation of N-terminal glutamine (both at peptide level) were set as variable modifications. Endoproteinase semi-Arg-C/P (semi Arg-C specificity with Arg-Pro cleavage allowed) was set as the enzyme, allowing for no missed cleavages. Mass tolerance was set to 10 ppm on the precursor ion and to 0.5 Da on fragment ions. Peptide charge was set to 1+, 2+ and 3+, and the instrument setting was switched to ESI-TRAP. Only peptides ranked the highest, had a minimum amino acid length of seven, scored above the threshold score set at 95% confidence, and belonged to the category of in vivo- or in vitro-blocked N-terminal peptides compliant with the rules of iMet processing [52] were withheld. More specifically, iMet processing was considered in the case of iMet-starting N-termini followed by any of Ala, Cys, Gly, Pro, Ser, Thr, Met or Val, and only if the iMet was encoded by ATG or any of GTG or TTG near-cognate start codons (Additional file 2: Table S17). While the occurrence of N-terminal protein acetylation (Nt-acetylation) and Nt-formyl retention are not trivial in bacteria (i.e. N-terminal protein acetylation and retention of the Nt-formyl group affected about 10% and 5% of uniquely identified protein in *E. coli*), the low degree of these N-terminal modifications at steady-state levels [53] – a finding in contrast to eukaryotic nascent protein N-termini – warrant caution to unequivocally assign bacterial protein N-termini as proxies of translation initiation. Because of the aforementioned reasons, we only selected Nt-formylated iMet-starting N-termini as a high confidence subset of TIS-indicative N-termini to experimentally validate the accuracy of the predictions (Additional file 2: Table S18).

### Assessing model accuracy

Sensitivity, specificity and positive predictive values were calculated from all genes supported by either high confidence n-terminal peptides (*S*. Typhimurium) or experimentally verified protein starts (*E. coli*). Supported predicted ORFs were considered true positives, whereas predicted ORFs that disagreed with supported positions were classified as false positives. False negatives were assigned from supported genes where no ORF was predicted. All in-frame cognate and near cognate start codons in stop-to-stop regions of supported genes that were neither predicted nor supported were considered true negatives.

### Further support for predicted ORFs

GC content was calculated at the third nucleotide positions for all annotated and predicted ORFs and mean GC values were summarised for each subgroup of predicted ORFs (matching annotations, truncations and extensions) in 9 nt sliding windows, over regions 57 nt upstream and 57 nt downstream of the annotated or predicted start sites.

Nucleotide sequences (–20 to + 20 nt) were extracted around the predicted and annotated TIS in the *S*. Typhimurium and *E. coli* genomes. Sequence logos were generated for each subgroup of matching annotations, truncations, extensions and novel genes, using the weblogo tool [54].

The minimum free energy of RNA secondary structure around predicted and annotated ORFs was estimated with RNAfold version 2.1.9 from the ViennaRNA package [48]. Mean free energy values were summarised for each ORF class in a 39-nt sliding window across regions 57 nt up and downstream of the start codon.

Read distributions were created for each subgroup of predicted ORFs (matching annotations, truncations, extensions and novel genes) and their corresponding annotated TIS. Distributions of ribo-seq reads adjusted to codon level resolution were summarised in regions 30 nt upstream and downstream of the first nucleotide of the initiation codon and total counts of each individual region were scaled to a sum of one in order to normalise profiles for differences in gene expression levels. Meta plots were then produced to show the proportion of reads over the window position from all predicted subgroups and their corresponding annotated start codons.

Amino acid sequences of novel ORF were compared to known proteins in the non-redundant protein database (Update date: 2016/12/15) and protein domains (cdd.v.3.15) using BLASTP [48, 55] (version 2.5.1+). Hits with the greatest coverage of query sequence and lowest e-value were selected. Hits were considered highly similar if they shared > 95% identity to a protein sequence over 100% of the novel ORF sequence.

### Statistical analysis

Wilcoxon rank sum tests were performed on ribo-seq distributions in Fig. 3a. The ratio of mean ribo-seq counts, per gene, upstream (positions –30 to –1 nt) or downstream (positions 0 to 29 nt) for extended and truncated positions were compared to matching positions using Wilcoxon rank sum test with continuity correction. Third codon GC distributions in Fig. 4b (upper) were also assessed with Wilcoxon rank sum tests with continuity correction, comparing the difference between the mean ribo-seq counts in downstream regions (positions 18 to 57 nt) and upstream regions (positions –57 to 18 nt), per gene, for extended or truncated and matching genes. Regions were selected to exclude the bias caused by SD and start codon sequence composition (peaks in the –18 to 6 nt regions).

## Additional files

Additional file 1: **Figures S1**–**S9. Figure S1.** Ribo-seq meta profiles at start and stop codons *S*. Typhimurium. **Figure S2.** Read length distributions at Shine–Dalgarno motifs. **Figure S3**: Codon-specific read length distributions. **Figure S4.** Additional prediction support. **Figure S5.** Third codon periodicity and GC content. **Figure S6.** Evidence for predicted novel translation initiation sites. **Figure S7.** Ribo-seq meta profiles at start codons for *E. coli*. **Figure S8.** Library read length distributions. **Figure S9.** Read length adjustments. (PDF 4700 kb)

Additional file 2: **Tables S1**–**S18. Table S1.** Variable importance in the *S*. Typhimurium monosome sample. **Table S2.** Variable importance in the *S*. Typhimurium polysome sample. **Table S3.** N-terminal support for *S*. Typhimurium predicted ORFs. **Table S4.** Predicted ORFs from the *S*. Typhimurium dataset. **Table S5.** Assessment of the contribution of parameter types to the predictive performance. **Table S6.** Support for novel predicted ORFs. **Table S7.** Ribo-seq sample info. **Table S8.** Variable importance in the *E. coli* TET2 sample. **Table S9.** Variable importance in the *E. coli* TET3 sample. **Table S10.** Variable importance in the *E. coli* Li1 sample. **Table S11.** Variable importance in the *E. coli* Li3 sample. **Table S12**: Variable importance in the *E. coli* Mohammad1 sample. **Table S13.** Variable importance in the *E. coli* Mohammad2 sample. **Table S14.** ORF predictions in the *E. coli* tetracycline libraries. **Table S15.** ORF predictions in the *E. coli* Li libraries. **Table S16.** ORF predictions in the *E. coli* Mohammad libraries. **Table S17.** Blocked N-terminal peptides. **Table S18.** High confidence N-terminal peptides. (XLSX 321 kb)

## Abbreviations

AUC: area under curve
CDS: protein coding sequence
FPKM: fragments per kilobase per million mapped reads
iMet: initiator methionine
nt: nucleotide
ORF: open reading frame
ribo-seq: ribosome profiling
SD: Shine–Dalgarno sequence
TIS: translation initiation site

## References

[1] Delcher AL, Harmon D, Kasif S, White O, Salzberg SL. Improved microbial gene identification with GLIMMER. Nucleic Acids Res. 1999;27:4636–41. 10.1093/nar/27.23.4636 PMC148753 10556321

[2] Brocchieri L, Kledal TN, Karlin S, Mocarski ES (2005). Predicting coding potential from genome sequence: application to betaherpesviruses infecting rats and mice. J Virol.

[3] Hyatt D, Chen G-L, Locascio PF, Land ML, Larimer FW, Hauser LJ (2010). Prodigal: prokaryotic gene recognition and translation initiation site identification. BMC Bioinformatics.

[4] Hall J, Hazlewood GP, Surani MA, Hirst BH, Gilbert HJ (1990). Eukaryotic and prokaryotic signal peptides direct secretion of a bacterial endoglucanase by mammalian cells. J Biol Chem.

[5] Kozak M (1999). Initiation of translation in prokaryotes and eukaryotes. Gene.

[6] Suzek BE, Ermolaeva MD, Schreiber M, Salzberg SL (2001). A probabilistic method for identifying start codons in bacterial genomes. Bioinformatics.

[7] Zhu H-Q, Hu G-Q, Ouyang Z-Q, Wang J, She Z-S (2004). Accuracy improvement for identifying translation initiation sites in microbial genomes. Bioinformatics.

[8] Ou H-Y, Guo F-B, Zhang C-T (2004). GS-Finder: a program to find bacterial gene start sites with a self-training method. Int J Biochem Cell Biol.

[9] Tech M, Morgenstern B, Meinicke P (2006). TICO: a tool for postprocessing the predictions of prokaryotic translation initiation sites. Nucleic Acids Res.

[10] Hartmann EM, Armengaud J (2014). N-terminomics and proteogenomics, getting off to a good start. Proteomics.

[11] Berry IJ, Steele JR, Padula MP, Djordjevic SP (2016). The application of terminomics for the identification of protein start sites and proteoforms in bacteria. Proteomics.

[12] Nakahigashi K, Takai Y, Kimura M, Abe N, Nakayashiki T, Shiwa Y (2016). Comprehensive identification of translation start sites by tetracycline-inhibited ribosome profiling. DNA Res.

[13] Ingolia NT, Ghaemmaghami S, Newman JRS, Weissman JS (2009). Genome-wide analysis in vivo of translation with nucleotide resolution using ribosome profiling. Science.

[14] Ingolia NT, Lareau LF, Weissman JS (2011). Ribosome profiling of mouse embryonic stem cells reveals the complexity and dynamics of mammalian proteomes. Cell.

[15] Ingolia NT, Brar GA, Stern-Ginossar N, Harris MS, Talhouarne GJS, Jackson SE (2014). Ribosome profiling reveals pervasive translation outside of annotated protein-coding genes. Cell Rep.

[16] O’Connor PBF, Li G-W, Weissman JS, Atkins JF, Baranov PV (2013). rRNA:mRNA pairing alters the length and the symmetry of mRNA-protected fragments in ribosome profiling experiments. Bioinformatics.

[17] Mohammad F, Woolstenhulme CJ, Green R, Buskirk AR (2016). Clarifying the translational pausing landscape in bacteria by ribosome profiling. Cell Rep.

[18] Bazzini AA, Johnstone TG, Christiano R, Mackowiak SD, Obermayer B, Fleming ES (2014). Identification of small ORFs in vertebrates using ribosome footprinting and evolutionary conservation. EMBO J.

[19] Han Y, Gao X, Liu B, Wan J, Zhang X, Qian S-B (2014). Ribosome profiling reveals sequence-independent post-initiation pausing as a signature of translation. Cell Res.

[20] Woolstenhulme CJ, Guydosh NR, Green R, Buskirk AR (2015). High-precision analysis of translational pausing by ribosome profiling in bacteria lacking EFP. Cell Rep.

[21] Chew G-L, Pauli A, Rinn JL, Regev A, Schier AF, Valen E (2013). Ribosome profiling reveals resemblance between long non-coding RNAs and 5’ leaders of coding RNAs. Development.

[22] Calviello L, Mukherjee N, Wyler E, Zauber H, Hirsekorn A, Selbach M (2016). Detecting actively translated open reading frames in ribosome profiling data. Nat Methods.

[23] Brar GA, Yassour M, Friedman N, Regev A, Ingolia NT, Weissman JS (2012). High-resolution view of the yeast meiotic program revealed by ribosome profiling. Science.

[24] Michel AM, Choudhury KR, Firth AE, Ingolia NT, Atkins JF, Baranov PV (2012). Observation of dually decoded regions of the human genome using ribosome profiling data. Genome Res.

[25] Crappé J, Van Criekinge W, Trooskens G, Hayakawa E, Luyten W, Baggerman G (2013). Combining in silico prediction and ribosome profiling in a genome-wide search for novel putatively coding sORFs. BMC Genomics.

[26] Pauli A, Norris ML, Valen E, Chew G-L, Gagnon JA, Zimmerman S (2014). Toddler: an embryonic signal that promotes cell movement via Apelin receptors. Science.

[27] Duncan CDS, Mata J (2014). The translational landscape of fission-yeast meiosis and sporulation. Nat Struct Mol Biol.

[28] Fritsch C, Herrmann A, Nothnagel M, Szafranski K, Huse K, Schumann F (2012). Genome-wide search for novel human uORFs and N-terminal protein extensions using ribosomal footprinting. Genome Res.

[29] Lee S, Liu B, Lee S, Huang S-X, Shen B, Qian S-B (2012). Global mapping of translation initiation sites in mammalian cells at single-nucleotide resolution. Proc Natl Acad Sci U S A.

[30] Nakahigashi K, Takai Y, Shiwa Y, Wada M, Honma M, Yoshikawa H (2014). Effect of codon adaptation on codon-level and gene-level translation efficiency in vivo. BMC Genomics.

[31] Heyer EE, Moore MJ (2016). Redefining the translational status of 80S monosomes. Cell.

[32] Martens AT, Taylor J, Hilser VJ (2015). Ribosome A and P sites revealed by length analysis of ribosome profiling data. Nucleic Acids Res.

[33] Hwang J-Y, Buskirk AR (2017). A ribosome profiling study of mRNA cleavage by the endonuclease RelE. Nucleic Acids Res.

[34] Shine J, Dalgarno L (1975). Determinant of cistron specificity in bacterial ribosomes. Nature.

[35] Nakagawa S, Niimura Y, Miura K-I, Gojobori T (2010). Dynamic evolution of translation initiation mechanisms in prokaryotes. Proc Natl Acad Sci U S A.

[36] Muto A, Osawa S (1987). The guanine and cytosine content of genomic DNA and bacterial evolution. Proc Natl Acad Sci.

[37] Bentele K, Saffert P, Rauscher R, Ignatova Z, Blüthgen N (2013). Efficient translation initiation dictates codon usage at gene start. Mol Syst Biol.

[38] Goodman DB, Church GM, Kosuri S (2013). Causes and effects of N-terminal codon bias in bacterial genes. Science.

[39] Del Campo C, Bartholomäus A, Fedyunin I, Ignatova Z (2015). Secondary structure across the bacterial transcriptome reveals versatile roles in mRNA regulation and function. PLoS Genet.

[40] Li G-W, Burkhardt D, Gross C, Weissman JS (2014). Quantifying absolute protein synthesis rates reveals principles underlying allocation of cellular resources. Cell.

[41] Zhou J, Rudd KE (2012). EcoGene 3.0.. Nucleic Acids Res.

[42] Archer SK, Shirokikh NE, Beilharz TH, Preiss T (2016). Dynamics of ribosome scanning and recycling revealed by translation complex profiling. Nature.

[43] Gawron D, Ndah E, Gevaert K, Van Damme P (2016). Positional proteomics reveals differences in N-terminal proteoform stability. Mol Syst Biol.

[44] Martin M (2011). Cutadapt removes adapter sequences from high-throughput sequencing reads. EMBnet J.

[45] Quast C, Pruesse E, Yilmaz P, Gerken J, Schweer T, Yarza P (2013). The SILVA ribosomal RNA gene database project: improved data processing and web-based tools. Nucleic Acids Res.

[46] Langmead B, Salzberg SL (2012). Fast gapped-read alignment with Bowtie 2. Nat Methods.

[47] Balakrishnan R, Oman K, Shoji S, Bundschuh R, Fredrick K (2014). The conserved GTPase LepA contributes mainly to translation initiation in Escherichia coli. Nucleic Acids Res.

[48] Lorenz R, Bernhart SH, Höner Zu Siederdissen C, Tafer H, Flamm C, Stadler PF (2011). ViennaRNA Package 2.0.. Algorithms Mol Biol.

[49] H2O.ai. http://h2o.ai/resources. Accessed 10 May 2017.

[50] Staes A, Impens F, Van Damme P, Ruttens B, Goethals M, Demol H (2011). Selecting protein N-terminal peptides by combined fractional diagonal chromatography. Nat Protoc.

[51] Van Damme P, Van Damme J, Demol H, Staes A, Vandekerckhove J, Gevaert K (2009). A review of COFRADIC techniques targeting protein N-terminal acetylation. BMC Proc.

[52] Frottin F, Martinez A, Peynot P, Mitra S, Holz RC, Giglione C (2006). The proteomics of N-terminal methionine cleavage. Mol Cell Proteomics.

[53] Bienvenut WV, Giglione C, Meinnel T (2015). Proteome-wide analysis of the amino terminal status of Escherichia coli proteins at the steady-state and upon deformylation inhibition. Proteomics.

[54] Crooks GE, Hon G, Chandonia J-M, Brenner SE (2004). WebLogo: a sequence logo generator. Genome Res.

[55] Altschul SF, Madden TL, Schäffer AA, Zhang J, Zhang Z, Miller W (1997). Gapped BLAST and PSI-BLAST: a new generation of protein database search programs. Nucleic Acids Res.

[56] Edgar R (2002). Gene Expression Omnibus: NCBI gene expression and hybridization array data repository. Nucleic Acids Res.

[57] Vizcaíno JA, Csordas A, Del-Toro N, Dianes JA, Griss J, Lavidas I (2016). 2016 update of the PRIDE database and its related tools. Nucleic Acids Res.

