# Supplementary material for: Ribosome signatures aid bacterial translation initiation site identification
Source: BMC Biol. 2017 Aug 30;15:76. doi: 10.1186/s12915-017-0416-0 (PMC5576327; doi:10.1186/s12915-017-0416-0)
Supplement: Supplementary file 1 — Figures S1–S9. Figure S1. Ribo-seq meta profiles at start and stop codons S. Typhimurium. Figure S2. Read length distributions at Shine–Dalgarno motifs. Figure S3: Codon-specific read length distributions. Figure S4. Additional prediction support. Figure S5. Third codon periodicity and GC content. Figure S6. Evidence for predicted novel translation initiation sites. Figure S7. Ribo-seq meta profiles at start codons for E. coli. Figure S8. Library read length distributions. Figure S9. Read length adjustments. (PDF 4700 kb) [file 12915_2017_416_MOESM1_ESM.pdf]

Supplemental information

Page

Supplementary_Fig_S1: Ribo-seq meta profiles at start and stop codons *S.* Typhimurium. 2

Supplementary_Fig_S2: Read length distributions at Shine Dalgarno motifs. 3

Supplementary_Fig_S3: Codon specific read length distributions. 4

Supplementary_Fig_S4: Additional prediction support. 4

Supplementary_Fig_S5: Third codon periodicity and GC content. 5

Supplementary_Fig_S6: Evidence for predicted novel translation initiation sites. 5

Supplementary_Fig_S7: Ribo-seq meta profiles at start codons *E. coli.* 6

Supplementary_Fig_S8: Library read length distributions. 7

Supplementary_Fig_S9: Read length adjustments. 7

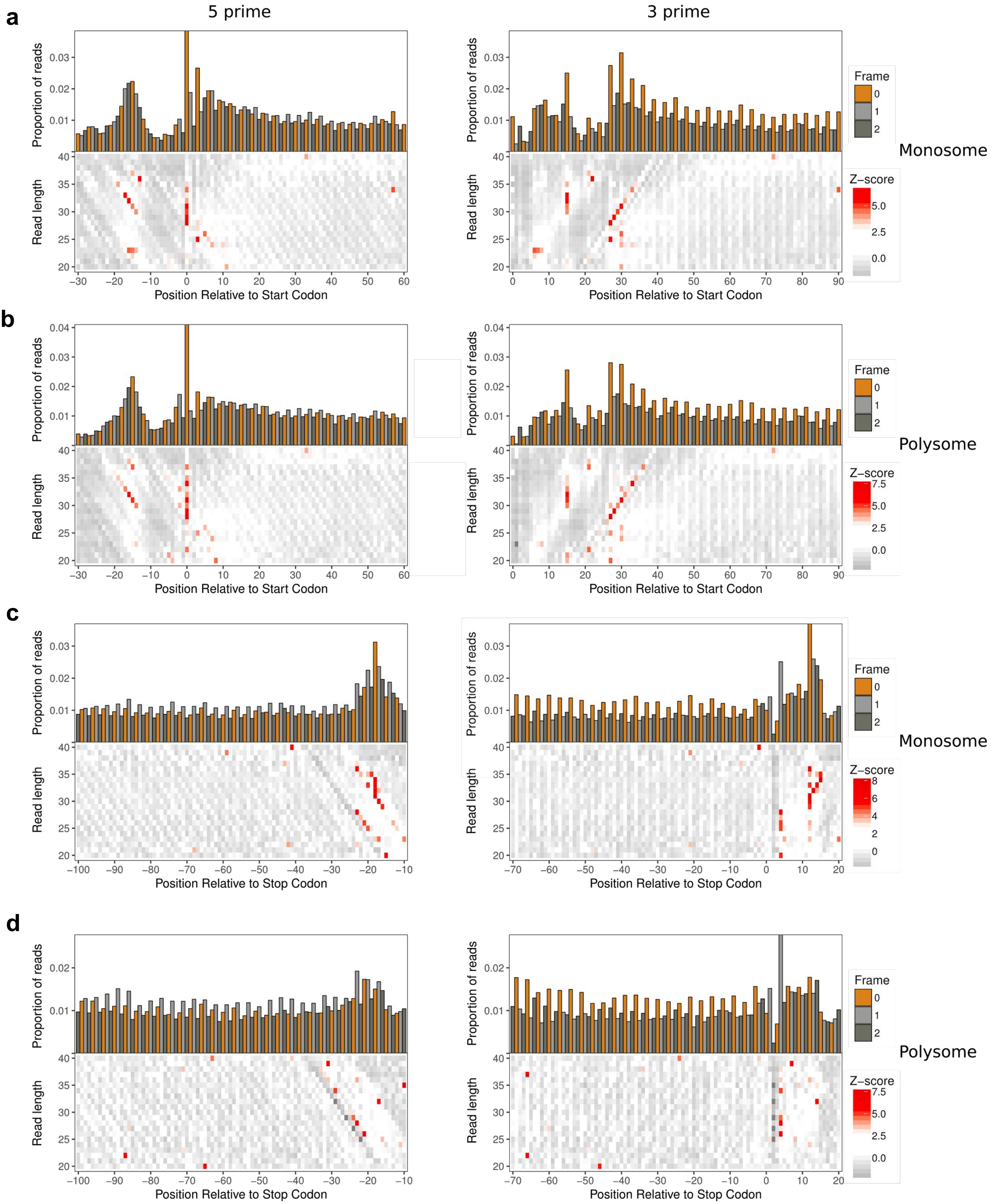

Supplementary_Fig_S1: Ribo-seq meta profiles at start and stop codons - *S.* Typhimurium
Ribo-seq meta-profiles in windows around start codons for annotated genes (n=4205) in the *S.* Typhimurium genome, contributions from each gene are scaled to a sum of one. (**upper**) Proportion of 5 or 3' ribo-seq read counts per nucleotide position, coloured by codon position. (**lower**) Heatmaps of 5' or 3' ribo-seq read counts per length, coloured by z-score per protected read length.

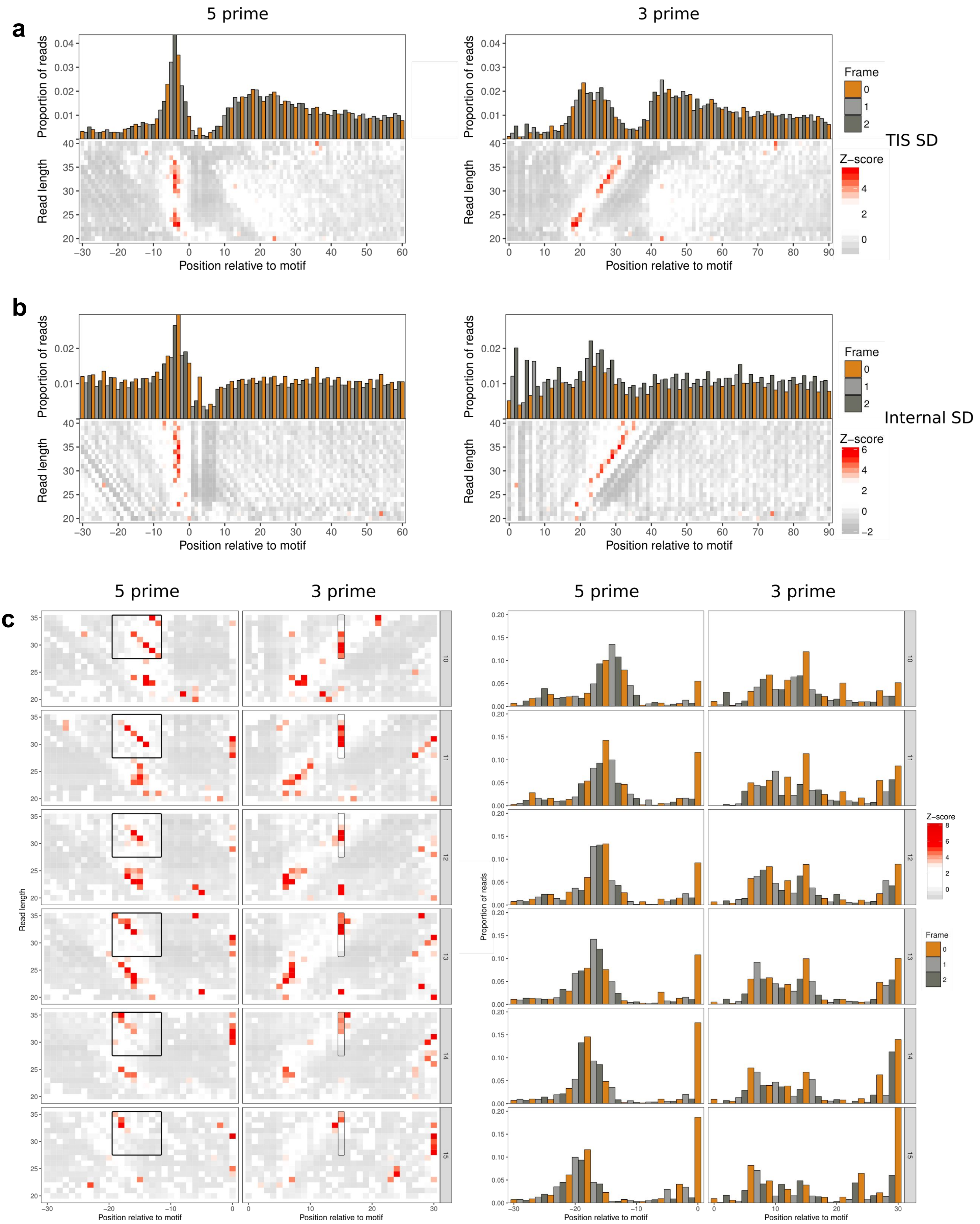

Supplementary_Fig_S2: Read length distributions at Shine Dalgarno motifs
Ribo-seq meta-profiles in windows around shine dalgarno sequences immediately upstream of a TIS (n=736) or at internal CDS positions (n=8564) in the *S.* Typhimurium genome, contributions from each motif window are scaled to a sum of one. Barcharts show the proportion of 5 or 3' ribo-seq read counts per nucleotide position, coloured by codon position. Heatmaps show 5' or 3' ribo-seq read counts per length, coloured by z-score per read length. (**a**) In relation to SD motifs upstream of initiation codons. (**b**) In relation to internal SD motifs within CDS regions. (**c**) In relation to SD motifs upstream of initiation codons, faceted by distance between the SD motif and initiation codon (nt).

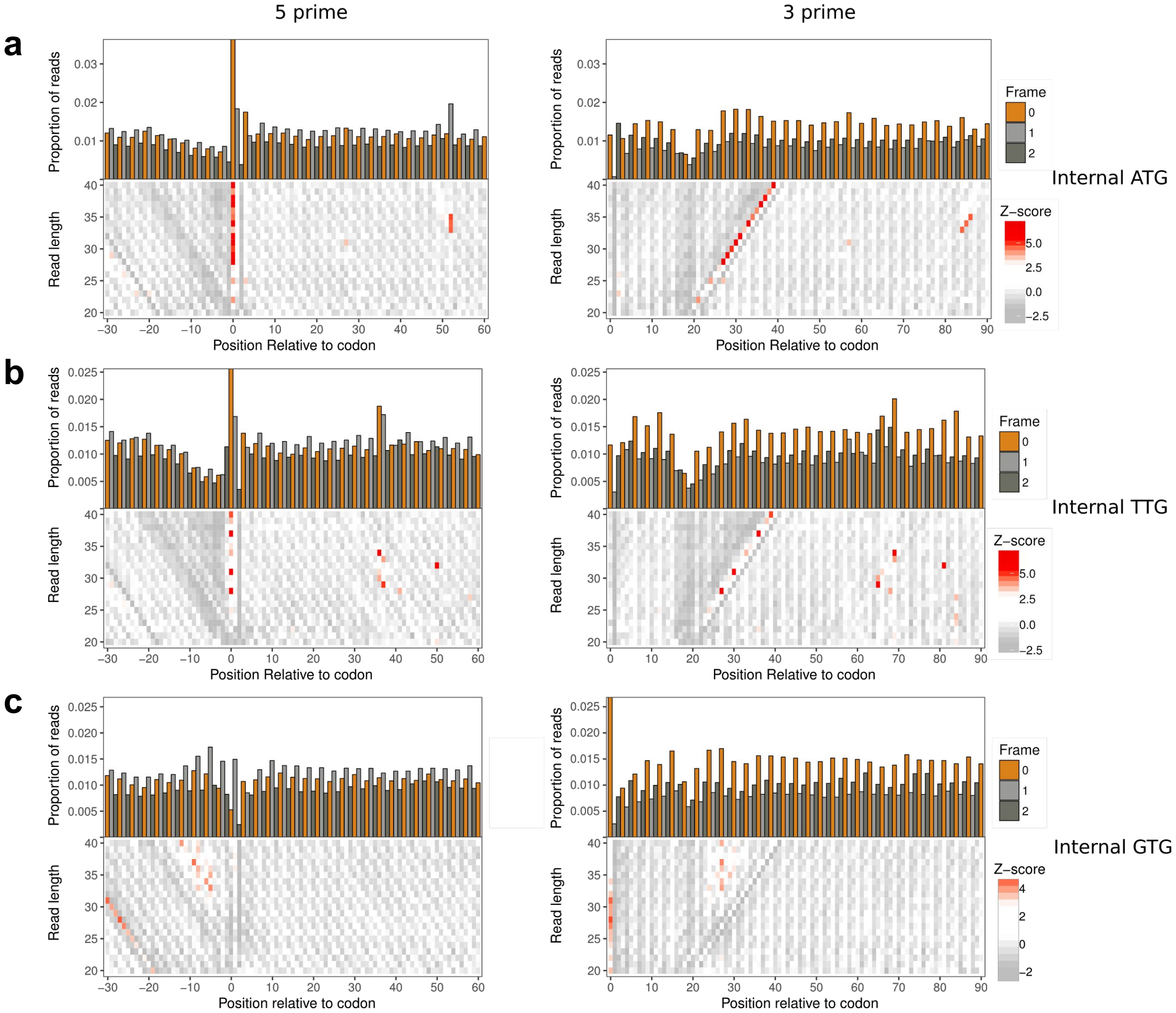

Supplementary_Fig_S3: Codon specific read length distributions.

S. typhimurium ribo-seq meta-profiles in windows around the most commonly used translation initiation codons at internal, in-frame CDS positions. Contributions from each codon window are scaled to a sum of one. (**upper**) Proportion of 5 or 3' ribo-seq read counts per nucleotide position, coloured by codon position. (**lower**) Heatmaps of 5' or 3' ribo-seq read counts per fragment length, coloured by z-score per read length. (**a**) ATG codons (n=35531). (**b**) GTG codons (n=35117). (**c**) TTG codons (n=17647).

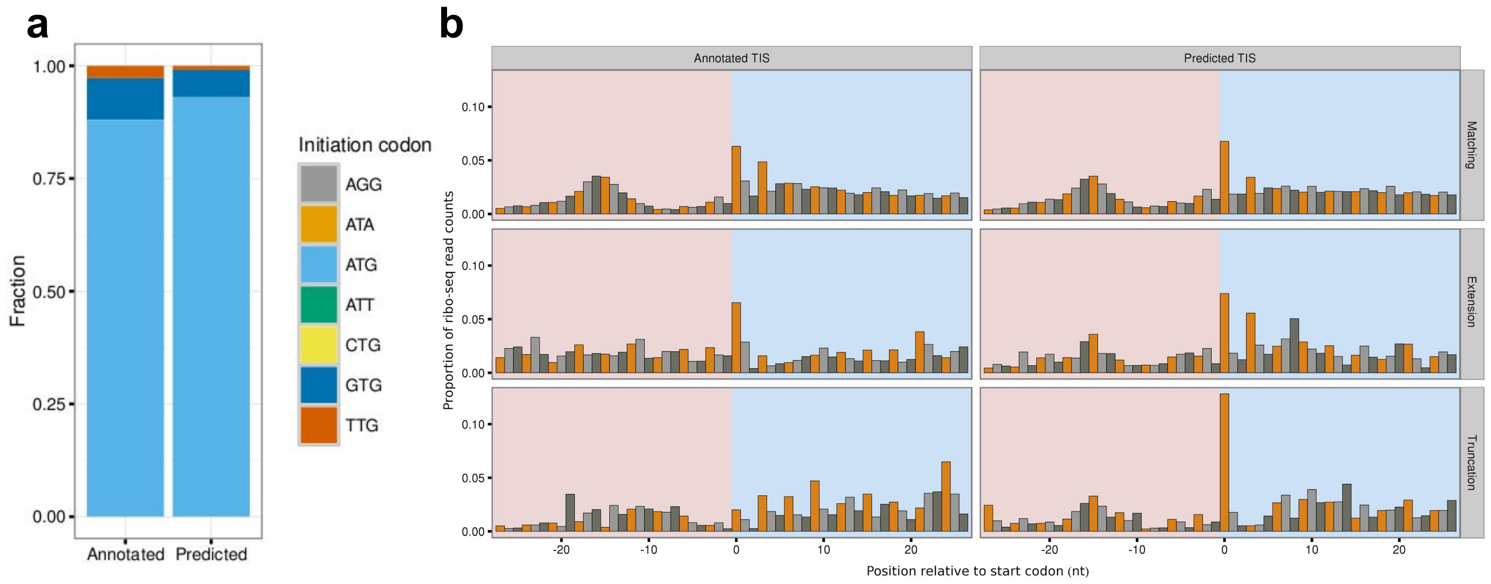

Supplementary_Fig_S4: Additional prediction support

(**a**) Showing the similarity in usage of different start codons between annotated (n=4653) and predicted (n=4334) *S.* Typhimurium ORFs. (**b**). Meta plots showing the proportion of scaled 5' ribo-seq read counts in relation to annotated or predicted translation initiation sites, for ORFs matching annotated genes (n=3853), predicted as extensions (n=214) or predicted as truncations (n=205), in the *S.* Typhimurium dataset. Contributions from each gene are scaled to a sum of one. Nucleotide positions are coloured by codon position. Upstream regions are highlighted in pink, downstream regions are highlighted in light blue.

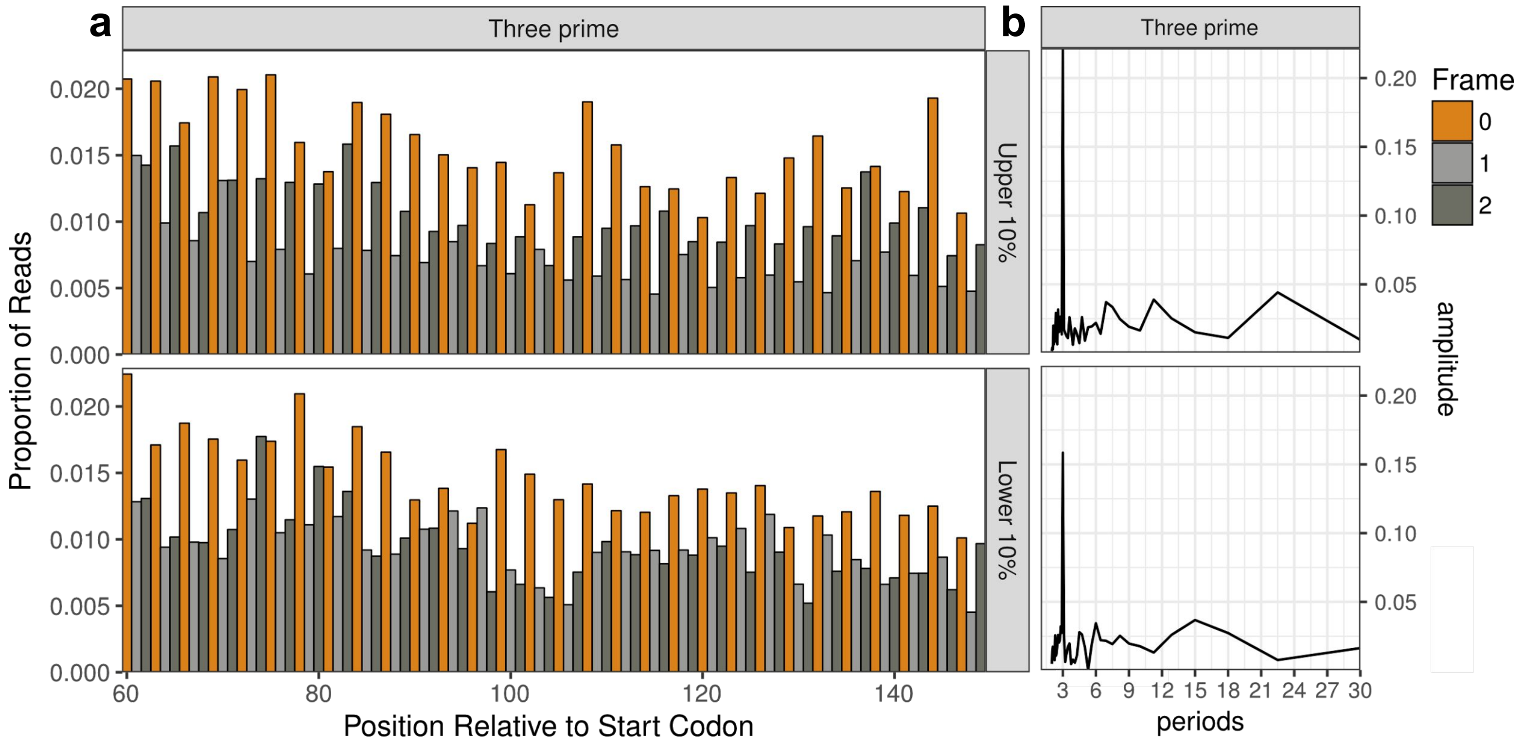

Supplementary_Fig_S5: Third codon periodicity and GC content.

(**a**) Proportion of 3' ribo-seq read counts per nucleotide position, from positions 60 - 150nt downstream of the annotated start codon in the *S.* Typhimurium genome, coloured by codon position. Contributions from each gene are scaled to a sum of one. (**upper**) the highest 10% of regions by third codon GC content (n=467). (**lower**) the bottom 10% of regions by third codon GC content (n=468). (**b**) fourier transform showing the periodicity in the distributions of (**a**).

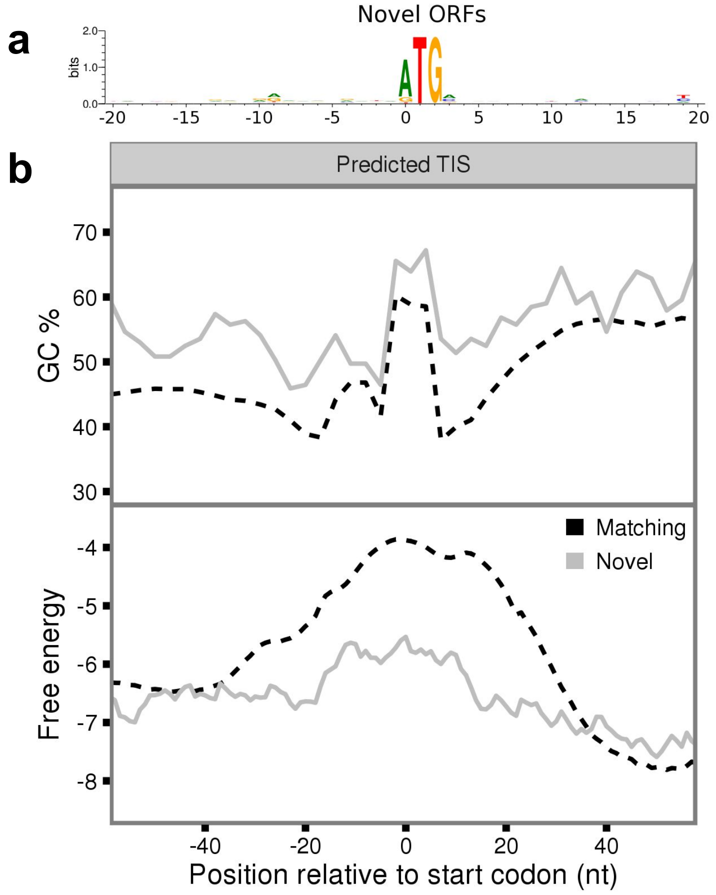

Supplementary_Fig_S6: Evidence for predicted novel translation initiation sites.

(**a**) Sequence motifs relation to predicted translation initiation sites (n=61), for sequences in all novel predicted ORFs in the *S.* Typhimurium dataset. (**B**) Meta-profiles in relation to annotated (n=3853) or predicted translation initiation sites. Black dotted lines representing ORFs matching annotated genes, grey lines represent novel predictions. (**upper**) Meta-profiles showing the percentage of GC content averaged in 9nt sliding windows, higher values downstream of the codon region are indicative of coding potential. (**lower**) Meta-profiles of free energy averaged in 39nt sliding windows, higher values represent a lower potential for secondary structure formation.

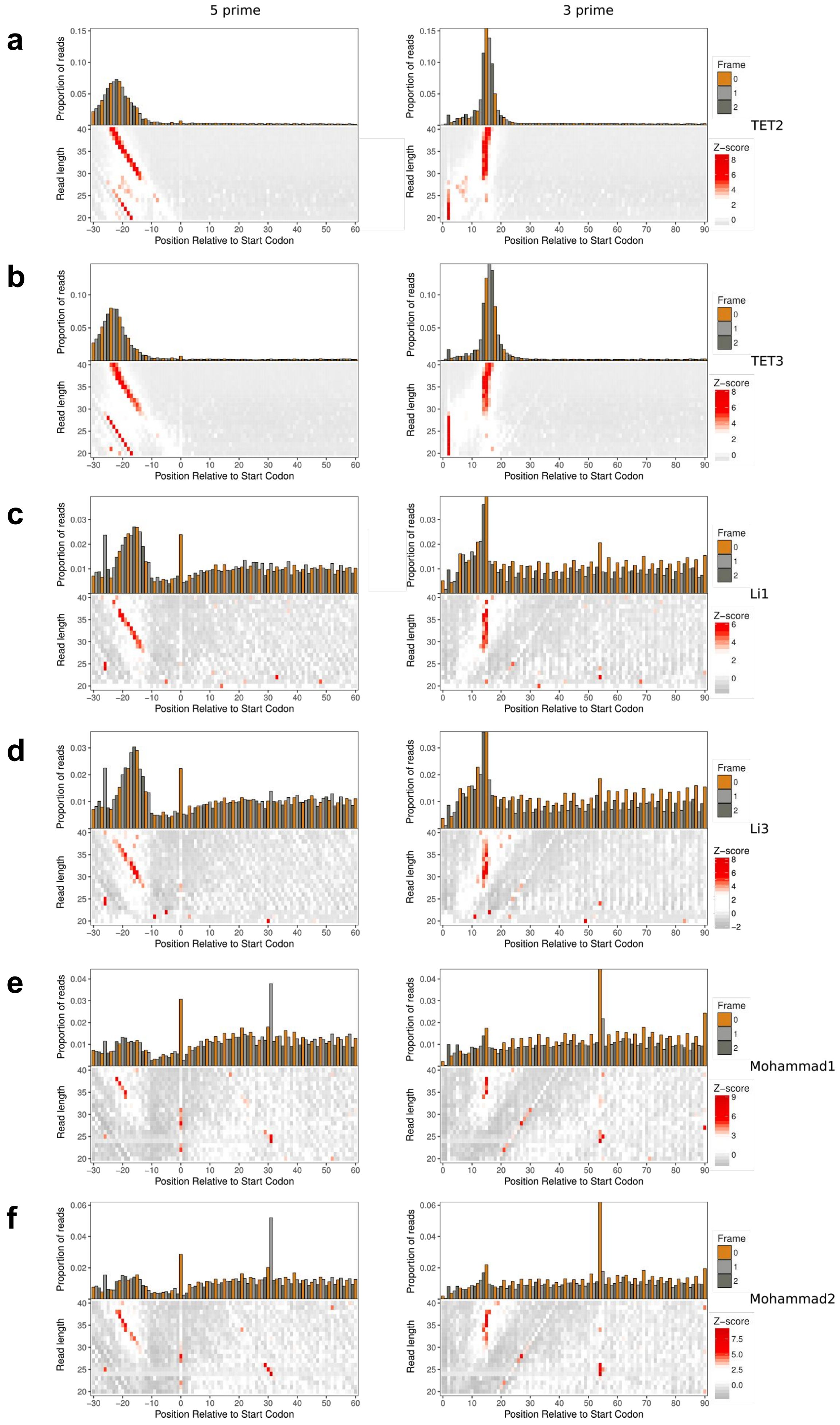

Supplementary_Fig_S7: Ribo-seq meta profiles at start codons - *E. Coli*
Ribo-seq meta-profiles in windows around start codons for annotated genes (n=3726) in the *E. coli* genome, contributions from each gene are scaled to a sum of one. (**upper**) Proportion of 5 or 3' ribo-seq read counts per nucleotide position, coloured by codon position. (**lower**) Heatmaps of 5' or 3' ribo-seq read length counts, coloured by z-score per read length.

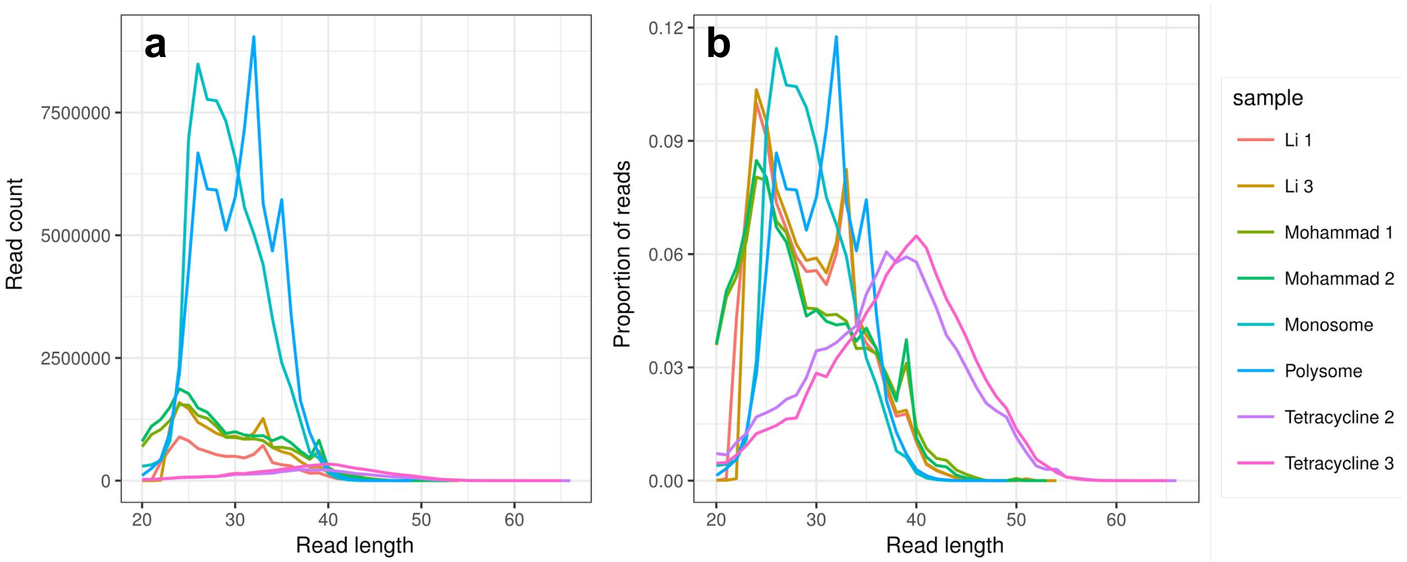

Supplementary_Fig_S8: Library read length distributions
Read length distributions of absolute (**a**) or proportional (**b**) counts of aligned ribo-seq reads per library.

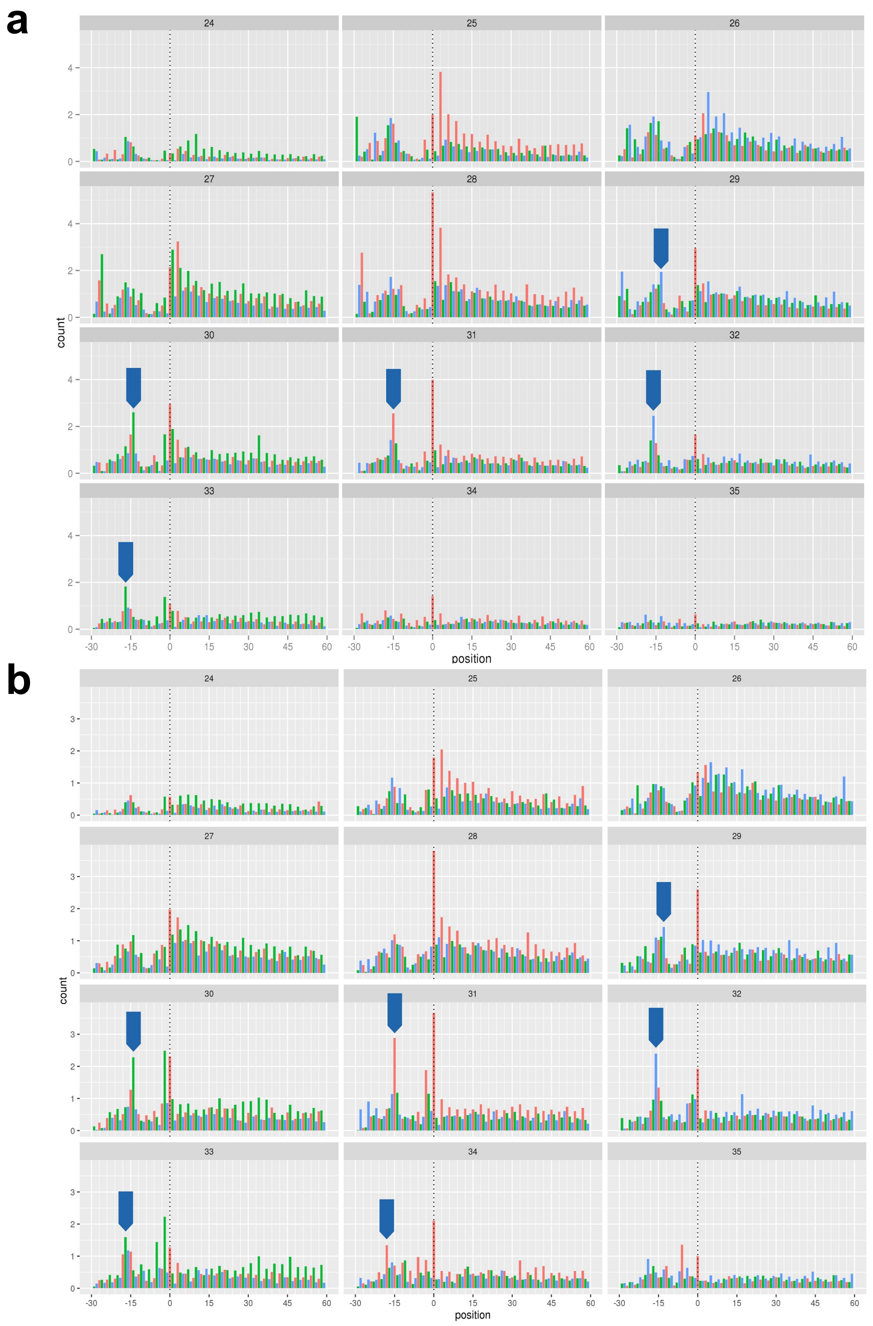

Supplementary_Fig_S9: Read lengths adjustments
The sum of scaled 5' ribo-seq counts from the (**a**) monosome or (**b**) polysome replicate, in -30 to +60nt windows around annotated start codons (n=4205) in *S.* Typhimurium, per read length, coloured by codon position. Contributions from each gene are scaled to a sum of one. Blue arrows indicate the peak corresponding to ribo-seq footprints translating the start codon.
